# Open-access synthetic spike-in mRNA-seq data for cancer gene fusions

**DOI:** 10.1186/1471-2164-15-824

**Published:** 2014-09-30

**Authors:** Waibhav D Tembe, Stephanie JK Pond, Christophe Legendre, Han-Yu Chuang, Winnie S Liang, Nancy E Kim, Valerie Montel, Shukmei Wong, Timothy K McDaniel, David W Craig, John D Carpten

**Affiliations:** Translational Genomics Research Institute (TGen), 445 N 5th Street, SUITE 600, Phoenix, AZ 85004 USA; Illumina, Inc, San Diego, CA USA

**Keywords:** RNA-seq, Gene fusions, Cancer genomics

## Abstract

**Background:**

Oncogenic fusion genes underlie the mechanism of several common cancers. Next-generation sequencing based RNA-seq analyses have revealed an increasing number of recurrent fusions in a variety of cancers. However, absence of a publicly available gene-fusion focused RNA-seq data impedes comparative assessment and collaborative development of novel gene fusions detection algorithms. We have generated nine synthetic poly-adenylated RNA transcripts that correspond to previously reported oncogenic gene fusions. These synthetic RNAs were spiked at known molarity over a wide range into total RNA prior to construction of next-generation sequencing mRNA libraries to generate RNA-seq data.

**Results:**

Leveraging *a priori* knowledge about replicates and molarity of each synthetic fusion transcript, we demonstrate utility of this dataset to compare multiple gene fusion algorithms’ detection ability. In general, more fusions are detected at higher molarity, indicating that our constructs performed as expected. However, systematic detection differences are observed based on molarity or algorithm-specific characteristics. Fusion-sequence specific detection differences indicate that for applications where specific sequences are being investigated, additional constructs may be added to provide quantitative data that is specific for the sequence of interest.

**Conclusions:**

To our knowledge, this is the first publicly available synthetic RNA-seq data that specifically leverages known cancer gene-fusions. The proposed method of designing multiple gene-fusion constructs over a wide range of molarity allows granular performance analyses of multiple fusion-detection algorithms. The community can leverage and augment this publicly available data to further collaborative development of analytical tools and performance assessment frameworks for gene fusions from next-generation sequencing data.

**Electronic supplementary material:**

The online version of this article (doi:10.1186/1471-2164-15-824) contains supplementary material, which is available to authorized users.

## Background

Oncogenic fusion genes underlie the mechanism of several common cancers and also constitute or encode important diagnostic and therapeutic targets. Fusions may drive oncogenic growth by joining a proliferation-inducing gene to an active promoter, by disrupting the function of tumor suppressor genes, or by creating novel functional products that rewire the biochemical pathways that regulate cellular division [1]. Research has led to identification of drugs that are currently used to target fusions in different malignancies. Examples include imatinib, tretinoin, and crizotinib, which target the *BCR-ABL*, *PML-RAR*, and *EML4-ALK* fusion products associated with chronic myelogenous leukemia [2, 3], acute promyelocytic leukemia [4–6], and non-small cell lung carcinoma [7–9], respectively. These established associations and clinical applications underscore the need to comprehensively and accurately detect fusions in cancer samples.

Next-generation sequencing technologies, particularly RNA sequencing (RNA-seq), have revealed an increasing number of recurrent fusions in a variety of cancers, and it is likely that their detection will have growing diagnostic and prognostic utility. As such, validating the laboratory and analysis methods to establish analytical parameters including the limit of detection, linearity, sensitivity, and specificity of fusion detection in tumor RNA specimens is critical for adoption in clinical research settings. For example, does a fusion transcript present at higher molarity (higher transcript abundance) correlate with higher number of fusion-supporting sequencing reads? Are there differences in detection algorithms’ efficacy with respect to specific fusion sequence and independent of abundance? Answering such questions and establishing robust metrics is difficult due to the lack of publicly available RNA-seq data specifically generated to capture gene fusions.

**Figure 1:**
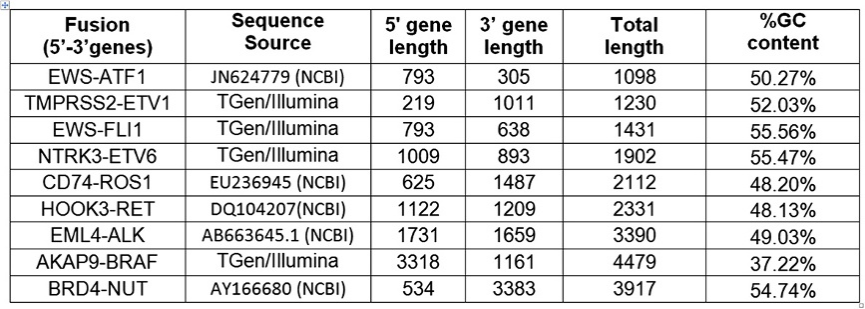
**Summary of nine synthetic fusion gene transcripts, excluding the poly-A tail.**

We have developed a set of nine synthetic poly-adenylated RNA transcripts that correspond to reported cancer fusion gene sequences (Figure 1 and Additional file 1: Table S1). These synthetic gene fusion RNA constructs (SGFRs) can be spiked at known concentrations into total RNA prior to mRNA library construction and barcoded to keep them separate from endogenous fusions. To demonstrate utility of these SGFRs, we performed a series of experiments and data analyses as described next.

## Methods

### Generation of synthetic gene fusion RNA (SGFR) constructs

**Figure 2:**
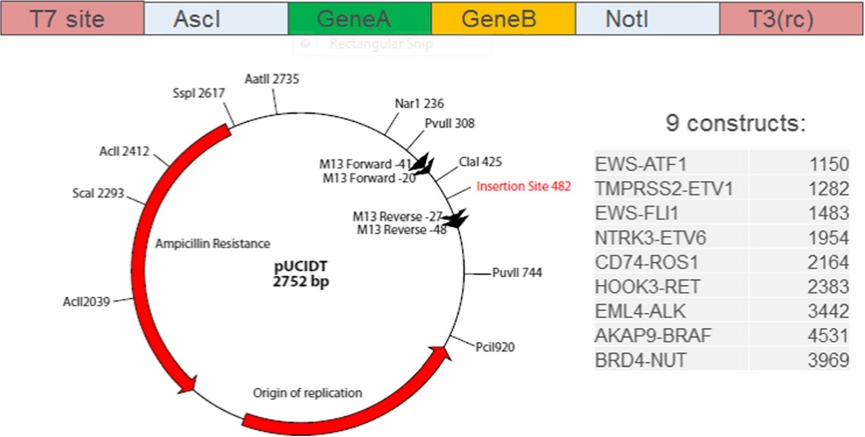
**Vector design: the gene sequence was synthesized by IDT and inserted into a pUCIDT vector.**

Sequences of nine transcripts containing oncogenic fusions were obtained from GenBank. Degenerate bases in the sequences were assigned a specific base and the final sequences can be found in the separate excel sheet. A T7 promoter sequence and AscI restriction enzyme site were added to the 5′ end of the sequence and a T3 and NotI sequence added to the 3′ end of the sequence to allow for linearization and transcription in both directions (Figure 2). The sequence was synthesized and inserted into a pUCIDT vector by IDT (San Diego, CA). Lyophilized plasmids were resuspended in 40 μL TE. 50 μL aliquots of Transformax™ EC100™ Chemically Competent E. coli (Epicenter, Madison WI) were thawed on ice and transfected with 1 μL (9.7-83.1 ng) of resuspended plasmid per the manufacturer’s suggested protocols. Transformed cells were plated on prewarmed 100 μg/mL ampicillin plates and incubated at 37°C overnight (18 hours). One colony from each plate was used to inoculate 5 mL LB broth (Teknova) containing 1× carbenicillin. Inoculated tubes were incubated overnight on a shaker at 37°C. Plasmids were isolated using the Qiagen Spin Miniprep Kit. The sequence of the purified plasmids were validated with Sanger sequencing. Purified plasmids were quantitated using the UV absorbance, then linearized with NotI-HF™ (New England Biolabs) at 37°C for 4 hours. Linearized plasmids were gel purified on a 0.8% agarose gel. Linear DNA was excised from the gels and purified using QIAquick Gel Extraction Kit and ethanol precipitated. DNA was transcribed to RNA using MegaScript® T7 Kit (Invitrogen) followed by poly(A) tailing using the Poly(A) Tailing Kit (Life Technologies) according to the manufacturer recommended protocols. Poly-A tailed RNA was cleaned up using MEGAclear™ Kit (Life Technologies, cat#AM1908) and ethanol precipitated in aliquots for long-term storage.

### RNA sequencing

RNA aliquots were washed in 70% ice cold ethanol, resuspended in 50 μL TE buffer (10 mM Tris–HCl pH 8.0, 1 mM EDTA), then quantitated using UV absorption. 2.2 ng of each RNA spike were pooled in a PCR plate, and the volume was brought up to 50 μL with RNase free water. A cDNA library was prepared using TruSeq Stranded mRNA LT Sample Prep Kit (Illumina®, cat# RS-122-2101) and sequenced on an Illumina MiSeq to confirm the sequences of the mRNA transcripts as a final QC step. Fresh aliquots of RNA were taken from storage, washed with 70% ice cold ethanol, resuspended in 1 × TE, and quantitated using RiboGreen (Invitrogen). RNA spikes were mixed together to create a high concentration pool with 40 nM of each spike. This pool was diluted and titrated into to 1 μg aliquots of COLO-829 total RNA (ATCC 1974). cDNA libraries were prepared using the TruSeq Stranded mRNA LT Sample Prep Kit (Illumina®, cat# RS-122-2101) following the manufacturer’s protocol. The resulting libraries were sequenced on the Illumina HiSeq2500 in Rapid Run mode using paired end reads with 101 cycles in each read.

In summary, equimolar amounts of all nine SGFRs were pooled together and this pool was titrated into total RNA from the melanoma cell line COLO-829 [10] at ten different abundances. Each SGFR abundance pool was prepared in duplicate. Libraries were prepared for sequencing using the Illumina TruSeq Stranded mRNA LT Sample Preparation Kit and sequenced on an Illumina HiSeq 2500 (2 × 101 cycles).

#### Bioinformatics

**Figure 3:**
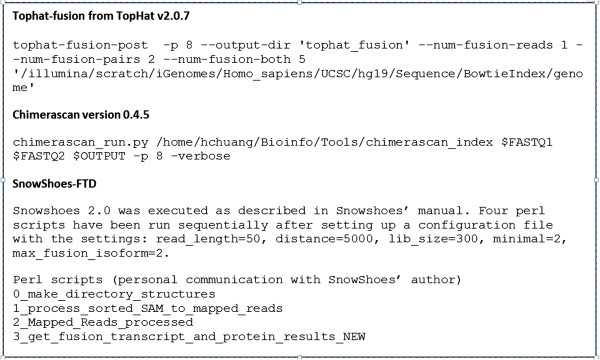
**Command-line parameters used for running the three fusion detection tools.** Reference genome was GRCh37. In each case, custom scripts were developed internally to extract statistics about fusion-supporting reads.

Illumina sequencing data was converted to FASTQ format using Casava pipeline followed by read quality assessment using FASTQC tool (http://www.bioinformatics.babraham.ac.uk/projects/fastqc/). We analyzed the data using three fusion detection tools: ChimeraScan [11], Tophat-Fusion [12], and Snowshoes-FTD [13] (hereafter referred as CHS, THF, and SSH respectively). The command-line parameters are described in Figure 3. For each analysis tool, we captured the number of sequencing reads supporting each of the nine SGFRs at various abundances (Additional file 1: Table S2), and this table was used for all subsequent analyses. In addition to the nine SGFRs, fusions endogenous to COLO829 were also detected by the analyses. We were able to confirm one endogenous fusion OIP5-NUSAP1 in independent wet-lab validation (Additional file 1: Table S3), although all callers did not identify it. Since endogenous fusions are out of scope of this study, they are not discussed further in this manuscript and we did not attempt to validate in wet-lab every predicted endogenous fusion. However, a parallel sample run with no SGFRs added showed zero reads mapping to the regions of select fused gene junctions, and therefore the COLO829 can be considered to be a high complexity neutral background sample for this study.

## Results and discussion

Analytically, gene fusions are typically detected from RNA-seq data by: 1) Aligning reads to a reference genome or transcriptome assembly; 2) Identifying discordant read pairs, i.e., pairs for which genomic distance between the two ends’ alignments is significantly different from the expected genomic distance based on library preparation; 3) Extracting split sections of the same read that align to different regions of the genome, thereby, indicating a potential fusion; 4) Algorithm-specific additional steps, such as contig construction, sequence homology search, guided analyses based on exon junction annotation files, etc.

We emphasize here that our focus is to demonstrate utility of the SGFR constructs for evaluating assay performance and to make them available to the clinical and research communities to further active research in gene-fusion detection methods. To that end, the choice of three representative algorithms and the analysis framework is based on our experience in analyzing such data. Since emphasis is on making RNA-seq gene fusion data publically available, we do not attempt to provide a detailed comparative assessment, pros-cons, or performance characterization of the growing number of gene fusion detection tools discussed elsewhere [14, 15]. However, to highlight the differences in the underlying analytical methods in these three fusion-detection tools, we briefly describe each of the approaches and direct readers to bibliography [11–13] for complete details. THF builds on Tophat to align RNA-seq reads using Bowtie [16] without using any annotation to independently align paired end reads, followed by segment mapping of unaligned reads that are used together for identifying candidate fusion junctions. Next, spliced fusion contig index is created and read segments are remapped using BLAST (in the *TophatFusionPost* step) followed by stitching all segments together into full read alignments that are further filtered based on criteria, such as number of fusion-supporting reads. SSH uses 50-bp reads that are aligned by BWA [17] guided by customized exon annotation file to identify potential fusions as well as unmapped reads. In our SSH analysis, we retained the first 50-bases from FASTQ files, and SnowShoes-FTD authors provided the annotation file (personal communication). Subsequent steps consists of using Megablast and a junction database to identify overlapping, spanning, and split reads to detect fusions that are further filtered using SnowShoes-FTD author provided false positive list. CHS uses known junctions from an annotation file that guides Bowtie alignment algorithm to find discordant read pairs and unmapped reads. Trimmed unmapped reads are aligned and used in conjunction with previous alignments to identify chimeric events by examining exon junctions from the annotation file. Thus, the three methods share an overall approach of identifying fusions based on aligning paired-end reads and detecting evidence of fusion junction. However, they are different with respect to the specific underlying alignment algorithm, read length, guidance from optionally provided annotation file, post-alignment processing to assemble fusion contigs, and parameters used to retain fusions from candidate fusions.

We also verified by running a separate parallel sample that the COLO-829 cell line provided a neutral background, i.e., it did not contain any of the nine SGFRs. Therefore, SGFRs in our experiment were not barcoded prior to spiking into the total RNA. However, barcoded SGFRs should be preferred in other cell lines to avoid mixing of spiked-in fusions and potential endogenous fusions.

**Figure 4:**
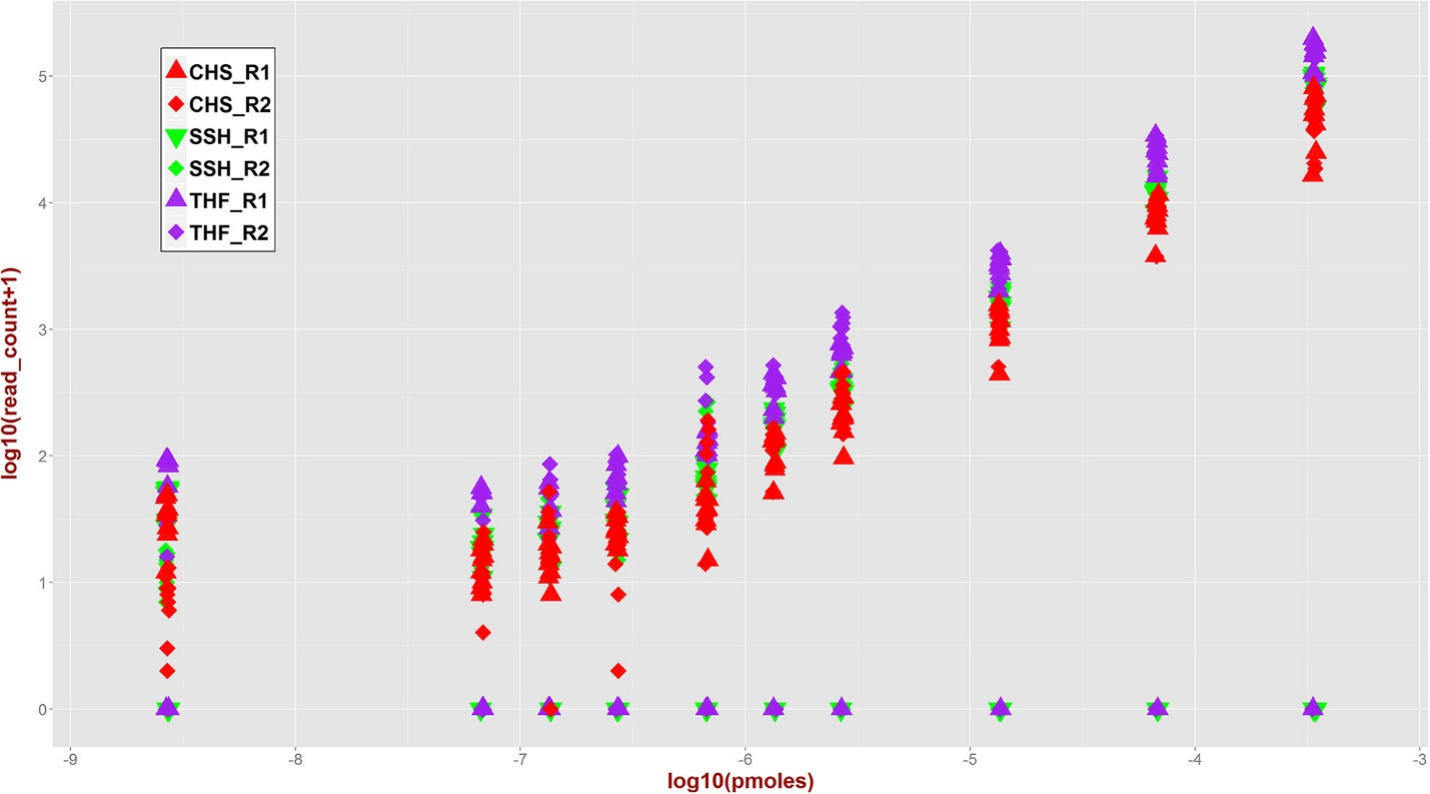
**Three algorithms TopHat-Fusion (THF), ChimeraScan (CHS), and SnowShoes-FTD (SSH) were used to identify and plot the number of fusion-supporting reads for SGFRs versus experimental input abundance.** Triangles correspond to data for sample replicate 1 (R1) and diamonds correspond to data for the second replicate (R2) with. Complete data is included as a table in supplementary materials.

**Figure 5:**
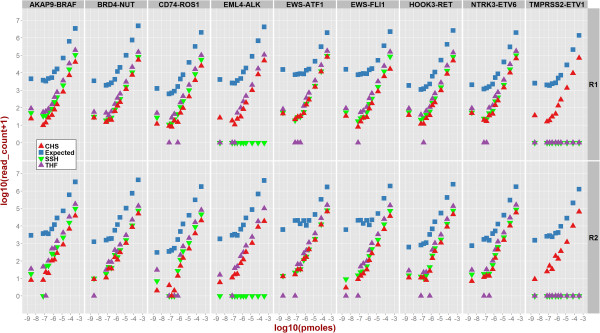
**To independently verify the presence of fusion reads (true positives) in the sequencing data, data was aligned using GSNAP to a combined reference sequence consisting of the human genome GRCh37 build and nine fusion transcripts.** For each fusion, the number of fusions supporting reads identified by GSNAP (blue squares), THF (purple triangle), CHS (red triangle), and SSH (inverted green triangle) are plotted for replicates R1 and R2.

Figure 4 demonstrates that at higher abundances, the relationship between number of detected fusions reads and abundance is linear. At lower abundances, the plateaued response might indicate high noise to signal ratio. To verify that fusion reads were present in the original data (true positive signal), we used GSNAP [18] as an independent tool to align entire data against a combined concatenated reference sequence consisting of human genome build GRCh37 and the nine synthetic fusions transcripts. Figure 5 shows the number of fusion-supporting reads identified by GSNAP (blue squares) along with those identified by the three gene fusion detection tools (triangles).

**Figure 6:**
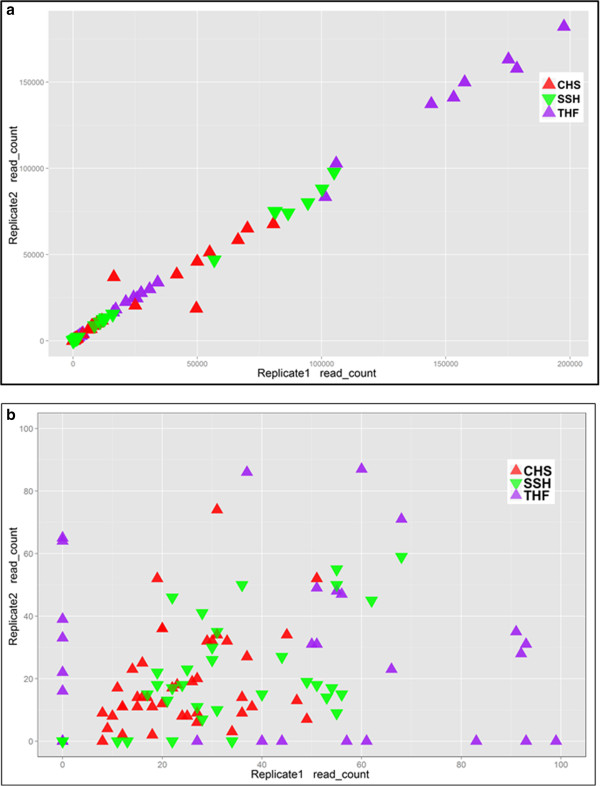
**Correlation between replicates based on number of fusion supporting reads.** Panel **(a)** shows fusion-supporting reads (X-axis: Replicate 1, Y-axis: Replicate 2) for high read count (>100). Pearson correlation was CHS: 0.9613, THF: 0.9990, SSH: 0.9986, All: 0.9955. Panel **(b)** shows data for low read count (<=100) with Pearson correlation values CHS: 0.3209, THF: 0.2577, SSH: 0.7292, All: 0.4025.

**Figure 7:**
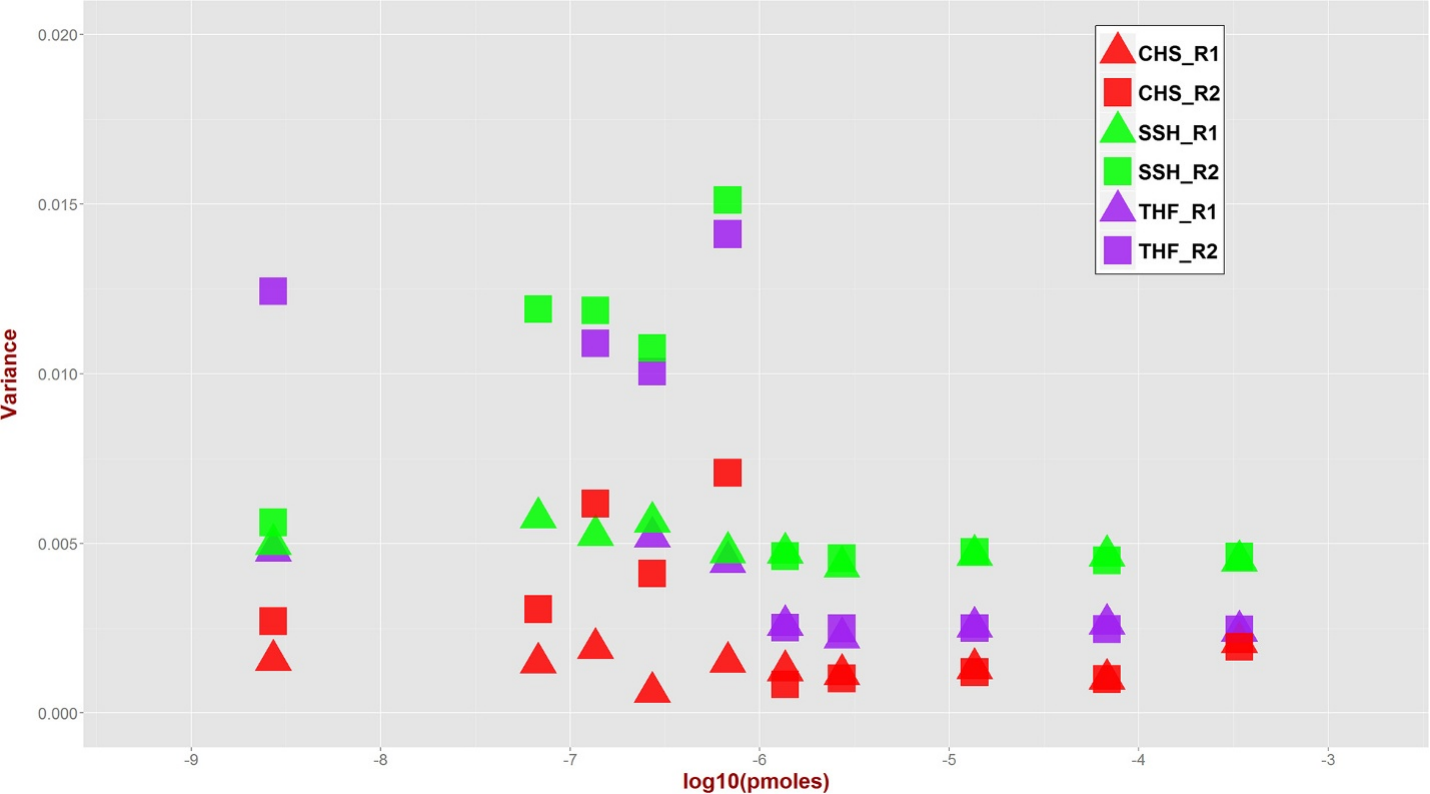
**Variance of fusion supporting reads across molarity.** For each fusion-transcript molarity (X-axis), variance of the fraction of fusion-supporting reads across nine fusions was calculated. Variances for replicates tend to be more similar at higher molarity indicating consistency in identifying fusion-supporting reads than at lower molarity.

To compare experimental replicates, we calculated the Pearson correlation between number of fusion-supporting reads between replicates (Figure 6) by dividing the data into high read count (>100) and low read count (<=100) groups chosen based on visual inspection of data for illustration purposes. For high read-counts, correlation between replicates’ reads for each tool as well as all reads combined together was high (CHS: 0.9613, THF: 0.9990, SSH: 0.9986, All: 0.9955). For low read-counts, corresponding correlation values were lower (CHS: 0.3209, THF: 0.2577, SSH: 0.7292, and All: 0.4025). This indicates higher difference between replicates at lower abundance values that should also translate to more differences in detected fusions at lower abundances. Figure 7 depicts the variability (Y-axis) in number of fusions reads against various abundances (X-axis). For each abundance, variance of the fraction of reads supporting each fusion from the total number of fusion-supporting reads was calculated when at least five out of nine, i.e., more than half, fusions had supporting reads. Clearly, at higher abundances (approximately 6 pMol or higher), variance is consistently low and replicates have almost equal variance indicated by overlapping data points.

**Figure 8:**
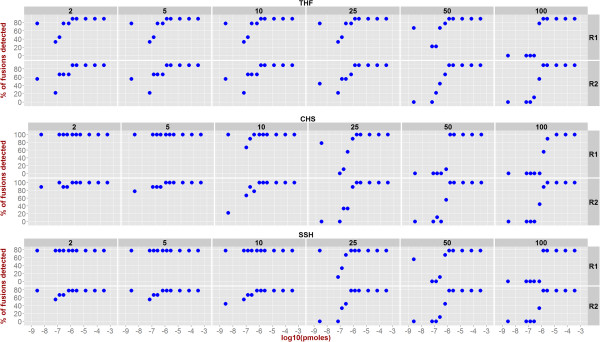
**Sensitivity of the three algorithms at various levels of fusion-supporting reads cutoff (2, 5, 10, 25, 50, and 100).**

To observe the effect of changing minimum number of reads required to call a fusion, Figure 8 depicts the number of fusions detected for each replicate at different minimum reads thresholds. Implicitly, Figure 8 also captures gene-fusion detection sensitivity as the ratio of number of detected fusions to the nine known fusions at various abundances for different minimum number of fusion-supporting reads threshold. For example, at 3.47 pMol, TophatFusion identifies all but the TMPRSS2-ETV1 fusion, with a sensitivity value of 8/9 = 88.88%. Sensitivity of replicates is highly similar, except for aberrations in the low abundance zones, and it consistently reaches high values at higher abundance. Since true negatives are unknown, specificity calculation is left as an open question.

**Figure 9:**
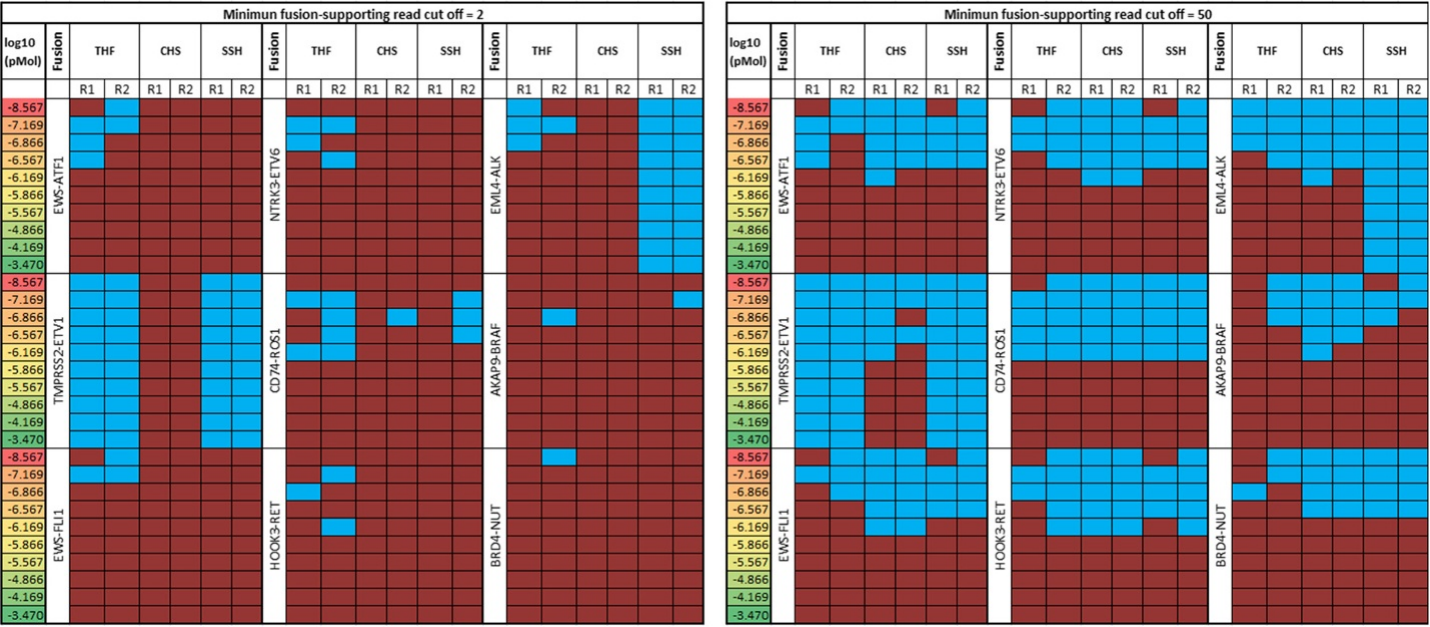
**Fusions detected by each algorithm.** For two example thresholds of 2 (left matrix) and 50 (right matrix) on minimum number of fusion-supporting reads, number of fusions detected at different concentrations for two replicates R1 and R2 are shown. Brown cell: fusion detected. Blue cell: fusion missed. For example, at minimum threshold of 2, BRD4-NUT was positively identified most frequently (59/60 times) and TMPRSS2-ETV1 was detected least frequently (20/60 times).

Figure 9 provides in a matrix form a more granular view of detected fusions (brown cells) and undetected fusions (blue cells) at example cut-offs of 2 and 50 fusion-supporting reads. At the minimum read threshold of 2 (Figure 9, left panel), a fusion was either detected or not detected in both replicates in 93% of the cases. BRD4-NUT (undetected in 1.5% cases) and TMPRSS2-ETV1 (undetected in 66% cases) marked the two extremes of detectability. None of the SGFRs was unambiguously detected across all molarities by all tools even at an extremely generous cut-off of minimum two fusion-supporting reads. This highlights the challenge in assessing performance metrics with a small set of synthetic constructs—even at the highest abundance in our experiments, 100% concordant results were not obtained for all of the SFGRs. The data are less reproducible at lower abundances. This indicates that for applications where specific fusion sequences are being investigated, additional constructs may be added to provide quantitative data that is specific for the sequence of interest.

Notably, some fusions were not detected by one or more tool(s) irrespective of molarity as shown by the points on X-axis in Figure 4. As shown in Figure 5, irrespective of the fusion transcript abundance all three tools detected EWS-ATF1, two tools detected EML4-ALK, and only one tool detected TMPRSS2-ETV1. On further investigation of SSH workflow, we discovered that fusion-supporting reads for both EML4-ALK and TMPRSS2-ETV1 were present in the initial candidate fusion list. However, these fusions were subsequently discarded by the SSH workflow when final list of fusions was reported. As end-users of the tool, we could not precisely identify specific reasons for this filtering out and a detailed investigation of SSH algorithm implementation is out of scope of this study. To explore why THF did not report TMPRSS2-ETV1 fusion, we extracted known fusion-supporting reads from GSNAP alignments and searched for those in the alignment files (generally known as *accepted_hits.bam*) generated by THF. We discovered that several fusion-supporting reads were aligned against TMPRSS2 (chr21:42.84-42.9 mb) and ETV1 (chr7:13.93-14.03 mb) loci across various molarities as shown in Additional file 1: Table S4. However, TMPRSS2-ETV1 fusion was not reported in the final list of fusions after the *TophatFusionPost* step was executed. A detailed investigation of actual THF algorithm implementation and specific reasons behind filtering out the fusion is out of scope of this study. However, observations based on additional investigation of unreported fusions highlight the critical importance of tool-specific criteria and parameters that might lead to false negatives or false positives—evidence for fusions from alignment data was processed differently by different tools yielding different results.

For the sake of completeness, we also note that each detection tool has a large number of input parameters that significantly affect its detection ability. Figure 4 depicts overall trend in capturing fusion-supporting reads based on our experimental design and chosen parameters. However, assessing the dynamic range and limits of detection for analytical tools will require extensive combinatorial selection of parameters, an in-depth analysis of algorithm implementation, and a much larger number of SGFRs across wide range of transcript abundance as part of testing and validation. These are out of scope of this study that is primarily focused on making available a publically available data for collaborative research and highlighting some of the issues in RNA-seq based gene fusion detection based on our analysis framework.

## Conclusion

The key contribution of this work is the first publicly available gene fusion RNA-seq data that specifically targets known oncogenic gene fusions that are gaining increasing importance in clinical genomics based on next-generation sequencing. The community can augment this dataset and the proposed analytical framework to further collaborative development of advanced analytical tools for gene fusion detection from RNA-seq data.

### Data availability

All sequencing data is available in FASTQ format from the Short Read Archive under accession number SRP043081.

## Electronic supplementary material

Additional file 1: Table S1: Fusion sequences. **Table S2.** Fusion read counts across all samples. **Table S3.** Endogenous fusions. **Table S4.** Fusion-supporting reads from TMPRSS2-ETV1 Tophat Fusion analysis. (XLSX 31 KB)
